# Characterizing the Association Between Asthma and Clinical Outcomes in Emergency Department Patients With Symptomatic COVID-19

**DOI:** 10.7759/cureus.79657

**Published:** 2025-02-25

**Authors:** Bachar Al Mazloum, Harriet Richardson, Yingwei Peng, Brian Rowe, Corinne M Hohl, Vlad Latiu, Dryden Chadwick, Kavish Chandra, Steven C Brooks

**Affiliations:** 1 Department of Public Health Sciences, Queen's University, Kingston, CAN; 2 Department of Public Health Sciences, Divisions of Cancer Care and Epidemiology and Canadian Cancer Trials Group, Cancer Research Institute, Queen's University, Kingston, CAN; 3 Department of Public Health Sciences, Department of Mathematics and Statistics, Division of Cancer Care and Epidemiology, Cancer Research Institute, Queen's University, Kingston, CAN; 4 Emergency Mecicine, University of Alberta, Edmonton, CAN; 5 Department of Emergency, Centre for Clinical Epidemiology and Evaluation, Vancouver Coastal Health Research Institute, University of British Columbia, Faculty of Medicine, Vancouver, CAN; 6 Department of Emergency Medicine, Queen's University, Kingston, CAN; 7 Department of Emergency Medicine, Dalhousie Medicine New Brunswick, Dalhousie, CAN; 8 Department of Emergency Medicine, Queen’s University, Kingston, CAN

**Keywords:** asthma, covid-19, emergency department, inhaled corticosteroids, observational study, severe acute respiratory syndrome coronavirus 2

## Abstract

Few studies have investigated the risks of developing intubation and death in patients seen in the emergency department (ED) with COVID-19 and pre-existing asthma. We conducted a retrospective cohort study using data from the Canadian COVID-19 Emergency Department Rapid Response Network (CCEDRRN) from March 1st, 2020, to December 31st, 2021. Inclusion criteria were age ≥18 and a positive SARS-CoV-2 test. The primary outcome was a composite of intubation or death, and the secondary outcome was severe COVID-19, as defined by the World Health Organization. Multivariable modified Poisson regression was used to assess the association between asthma and outcomes, adjusted for possible confounding. Out of 38,139 patients, 2,826 (7.41%) had asthma, and 17.1% were using inhaled corticosteroids (ICS). The study found no significant evidence suggesting an association between asthma and intubation or death in the hospital (relative risk (RR): 0.97; 95% CI: 0.86-1.1). The highest risk group for the primary outcome was patients aged 80+ years (RR: 10.54; 95% CI: 7.01-15.85), compared to the reference group 18-29 years. Users of ICS agents had a slightly higher risk of the primary outcome compared to non-ICS users (RR: 1.12; 95% CI: 1.01-1.25).

## Introduction

The severe acute respiratory syndrome coronavirus 2 (SARS-CoV-2) causes the clinical syndrome of coronavirus disease 2019 (COVID-19) [1]. On March 11, 2020, the World Health Organization (WHO) declared a global pandemic. According to the WHO, approximately 775 million cases of COVID-19 and 7 million related deaths were detected globally as of January 2024 [2]. The Public Health Agency of Canada reported approximately 4.9 million cases of COVID-19 as of January 2024 [3]. The global social and economic impacts of COVID-19 have been significant. For example, the unemployment rate has surged in Canada to 9.6%, with 3.1 million Canadians reporting job loss or reduced working hours due to the pandemic [4].

Asthma is a respiratory disease characterized by recurrent wheezing, shortness of breath, and coughing due to chronic inflammation of the airways [5]. The severity of the symptoms corresponds to encounters with triggers such as allergens, upper respiratory tract viral infections, and air particulate matter [5]. Over the last 20 years, the global prevalence of asthma has been steadily increasing, with 235 million cases worldwide [6]. For example, in Canada, approximately 3 million people (8.1%) have asthma, including 2.2 million adults and 80,000 children [7]. The United States of America experiences a higher prevalence with 14.9% of the population aged 12 years and older having a diagnosis of asthma.

During the early phases of the pandemic, recommendations from the Centers for Disease Control (CDC), the WHO, and the Global Initiative for Asthma (GINA) identified asthma as a risk factor for more severe COVID-19 [8-10]. The evidence on which these recommendations were based, however, was limited and often conflicting. Few studies have assessed the risk for poor outcomes after COVID-19 in patients with pre-existing asthma using real-world evidence outside of clinical trials.

Previous studies have debated the role of asthma in COVID-19 severity, with some suggesting an increased risk due to chronic airway inflammation and immune dysregulation, while others propose a protective effect linked to angiotensin-converting enzyme (ACE-2) receptor downregulation. Understanding these contrasting mechanisms is crucial for accurately assessing risk in asthmatic populations. The objective of our study was to assess the relationship between reported history of asthma, severe COVID-19 disease, and poor outcomes among patients presenting to the emergency department (ED) with symptomatic COVID-19. We hypothesized that asthma might have a protective effect, especially in those whose management included inhaled corticosteroids (ICS), given prior evidence that inhaled budesonide is associated with earlier recovery and reduced acute care visits [11]. This article was initially published as a preprint on the Research Square platform [12]. Clinical Trial Registration number is NCT04702945.

## Materials and methods

This was a retrospective cohort study of patients enrolled from March 1st, 2020, to December 31st, 2021, in the Canadian COVID-19 Emergency Department Rapid Response Network (CCEDRRN). The CCEDRRN study enrolled consecutive eligible patients presenting to EDs in 50 hospitals located in eight of ten Canadian provinces, including the four most populous ones (https://canadiancovid19ednetwork.org/) [13]. Participating EDs included those in both urban and rural regions within Canada (Supplementary Table 4). Researchers captured data on patients who presented to participating EDs from May 15, 2020, onwards until December 30, 2021. In addition to SARS-CoV-2 status, the CCEDRRN registry includes ED patient data from the index ED visits and, where applicable, hospital admission data [13]. The study’s inclusion criteria required participants to be part of the CCEDRRN cohort with suspected or confirmed COVID-19, aged 18 years or older, with a confirmed positive COVID-19 test, and exhibiting symptoms consistent with COVID-19. Exclusion criteria included individuals younger than 18 years (n = 5,459), those with a negative COVID-19 test (n = 138,967), and individuals who tested positive for COVID-19 but showed no symptoms (n = 12). This selection process ensured that only symptomatic COVID-19-positive adults were included in the final analysis. After applying these criteria, the final sample size for analysis was (n = 38,139) symptomatic COVID-19-positive adults.

Trained site-based research assistants collected data retrospectively from electronic and paper-based ED and hospital charts remotely or in an administrative area away from patient care into a central, web-based REDCap database (Vanderbilt University; Nashville, TN, USA) hosted at the study coordinating center. Research assistants captured demographics (e.g., age, sex, postal code), symptoms, clinical variables (e.g., arrival mode, time of day, severity as measured by the Canadian Triage and Acuity Scale (CTAS), vital signs), health history (e.g., medications, comorbid conditions that are included in the Clinical Characterization Protocol from the WHO-supported International Severe Acute Respiratory and Emerging Infection Consortium (ISARIC) [14], COVID-19 details (e.g., timing of infection, exposure risk variables, hospital diagnostic test results, COVID-19 test results), ED investigations (e.g., complete blood count, electrolytes, chest radiography, advanced imaging, etc.), treatments (e.g., intubation, medications), and patient outcomes (e.g., death, discharge home). The study developed rigorous governance policies, including a comprehensive data quality standard. This included regular data quality audits and logic checks integrated into REDCap. Site-level data verification was done on a subset of database entries with double data entry and source document verifications for nonsensical or outlying values.

The CCEDRRN registry was approved by the research ethics boards of all participating institutions under a waiver of informed consent for patient enrollment. The CCEDRRN registry was approved by the University of Calgary Conjoint Health Research Ethics Board (REB20-0534), the University of British Columbia Clinical Research Ethics Board (H20-01015), the University of Manitoba Health Research Ethics Board (H2020:261), Horizon Health Network Research Ethics Board (100890), Nova Scotia Health Authority Research Ethics Board (1025682), Queens University Health Sciences & Affiliated Teaching Hospitals Research Ethics Board (2165), Centre intégré de santé et de services sociaux de Chaudière-Appalaches (MP-23-2021-766), and the University of Saskatchewan Biomedical Research Ethics Board (1935) (Supplementary Table 5). This analysis was conducted within the scope of that approval. Model development and reporting followed TRIPOD standards [15]. Funders had no role in the collection, analysis, or interpretation of the data, the writing of the manuscript, or the decision to submit for publication. All methods were performed in accordance with the relevant guidelines and regulations.

Data variables

The primary exposure of interest was a pre-existing diagnosis of asthma. A diagnosis of asthma was recorded by research staff if it was self-reported by patients and documented in the ED triage note, documented in the medical record, or used as a diagnostic code. Validation of the diagnosis was not possible. We defined our primary study outcome as a composite of intubation or death in the hospital (including the ED). Our secondary outcome was severe COVID-19, defined by the WHO as: i) oxygen saturation is <90% on room air; ii) respiratory rate >30 breaths/min; iii) presence of any of the severe respiratory distress signs (accessory muscle use, inability to complete full sentences, grunting, central cyanosis and or presence of any of the general danger signs (such as inability to breastfeed, drink, or breath, lethargy or unconsciousness, and convulsions) [16]. We defined intubation as patients who had an endotracheal tube (ETT) inserted into their trachea in the ED, hospital ward, or ICU. An ED or in-hospital death was defined as death in one of those areas within 30 days of the index visit. All trade and generic drug names recorded by research staff and potential misspellings were reviewed to determine the use of ICS monotherapy and/or combination agents containing ICS agents. Information from some sites included pharmacy information records; however, patient compliance and adherence with inhaler therapy were not captured.

We used data from the medical record and telephone follow-up to collect patients’ vaccination status. If the two were discrepant, we coded the patient’s vaccination status according to their telephone follow-up data. For those patients with unknown vaccination status, after considering these data sources, we coded all patients who presented to the ED in 2020 as unvaccinated because the first COVID-19 vaccination was licensed for use by Health Canada on December 14, 2020. For patient encounters after 2020 with unknown vaccination status, we coded their immunization status according to their status as a health care provider, age, province of residence, and the date of the ED visit. Because vaccine eligibility in Canada was based on these factors and delivery was tightly controlled by public health authorities, we were able to use this rule with confidence to code patients as unimmunized when they were not eligible at the time of their ED visit. Patients who could not be coded as unimmunized using this rule were coded as having missing data regarding vaccination status during all aspects of the analysis.

We used a modified Charlson comorbidity index (CCI) adapted from the original CCI to represent the relative comorbidity of patients included in our analysis (Supplementary Table 6) [17,18]. Each comorbidity was assigned a weight from 1 to 6 based on the adjusted risk of mortality or resource use [18]. A patient having a score of zero indicates the absence of comorbidities, and higher scores indicate multiple comorbidities, higher chances of predicted mortality, and resource use. Eleven of the original CCI comorbidities were captured in CCEDRRN data used for our study: 1) coronary artery disease (CAD); 2) congestive heart failure (CHF); 3) chronic obstructive pulmonary disease (COPD); 4) chronic kidney disease; 5) diabetes mellitus; 6) mild liver disease 7) moderate/severe liver disease; 8) dementia; 9) active malignant neoplasm; 10) past malignant neoplasm; and 11) stroke, or transient ischemic attack. It should be noted that asthma was not included in the modified CCI. Each factor was given a score of 0 (absent) or 1 (present).

Statistical analysis

Categorical data were summarized and reported as percentages, tested with the statistical test, and all reported p-values were based on non-missing data. Continuous data from non-normally distributed variables were summarized as medians with interquartile ranges (IQR). Continuous variables were analyzed with a two sample T-test or Kruskal-Wallis test, where appropriate. We used a multivariate modified Poisson regression model to measure the association between asthma and the outcomes of interest among eligible patients. We chose candidate predictor variables based on literature review, clinical knowledge, and their availability within the CCEDRRN registry. They included demographics such as age and sex, ED variables such as mode of arrival, vital signs recorded at triage, COVID-19 symptoms, comorbidity, vaccination status, and use of ICS agents. Statistical analyses were performed using SAS version 9.4 software (SAS Institute Inc., Cary, NC), and statistical significance was inferred as p<0.05.

## Results

**Figure 1:**
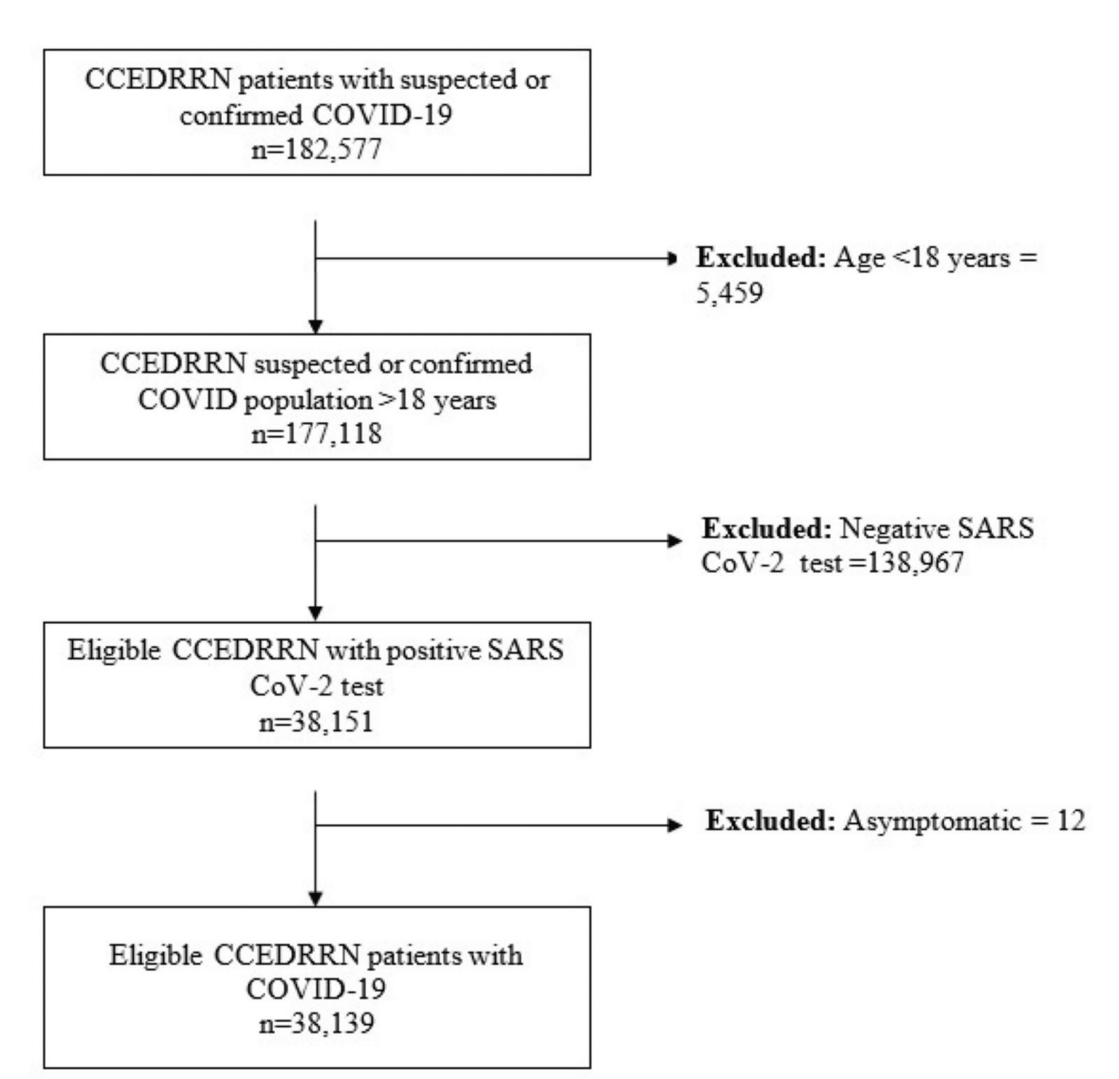
A flowchart demonstrating the selection of patients from the Canadian COVID-19 Emergency Department Rapid Response Network Database for this study to determine the relationship between asthma and COVID-19 outcomes. CCEDRRN: Canadian COVID-19 Emergency Department Rapid Response Network; SARS-CoV-2: severe acute respiratory syndrome coronavirus 2 The image was drawn by the author of this article using Microsoft Word. It is an original figure and has not been copied from any other source.

From March 1st, 2020, to December 31st, 2021, 182,577 patients were enrolled in the CCEDRRN registry. Of those, 38,139 were included in this study cohort (Figure 1). Of those, 18,263 (47.9%) were female, 27,985 (73.4%) were unvaccinated, and 2,826 (7.4%) patients had a history of asthma. Patient characteristics and outcomes are shown in Table 1. After adjusting for age, sex, ICS, presence of comorbidities, and vaccination status, we did not observe a significant association between asthma and the composite primary outcome of intubation or in-hospital death (RR: 0.97; 95% CI: 0.86, 1.1)(Table 2). Being male, older age, using ICS, having higher comorbidity, and being unvaccinated were factors independently associated with a higher frequency of intubation or in-hospital death. Similarly, we did not observe a significant association between asthma and the development of severe COVID-19 (RR: 0.96; 95% CI: 0.81, 1.13) (Table 3). Users of ICS agents had a slightly higher risk of the primary outcome compared to non-ICS users (RR: 1.12; 95% CI: 1.01, 1.25).

**Table 1:** Characteristics of patients included in the study by asthma status. ED: emergency department; ICS: inhaled corticosteroids; LABA: long-acting beta-agonist; LAMA: long-acting muscarinic agonist; SABA: short-acting beta-agonist **p-values in this table test whether there is a statistically significant difference in the distribution of each categorical variable between patients with asthma and those without asthma.

	All N=38,139	Asthma N=2,826 (7.4%)	No asthma N=35,313 (92.5%)	P-value**
Sex n (%)				<0.0001
Male	19,876 (52.1%)	1,198 (42.4%)	18,678 (52.9%)	
Female	18,263 (47.9%)	1,628 (57.6%)	16,635 (47.1%)	
Age category n (%)				0.033
18-29 years	3,998 (10.5%)	280 (9.9%)	3,718 (10.5%)	
30-39 years	5,480 (14.4%)	426 (15.0%)	5,054 (14.3%)	
40-49 years	6,356 (16.7%)	481 (17.0%)	5,875 (16.6%)	
50-59 years	6,787 (17.8%)	542 (19.2%)	6,245 (17.7%)	
60-69 years	5,726 (15.0%)	414 (14.7%)	5,312 (15.0%)	
70-79 years	4,626 (12.3%)	352 (12.5%)	4,274 (12.1%)	
>80+ years	5,166 (13.6%)	331 (11.7%)	4,835 (13.7%)	
Mode of arrival n (%)				<0.0001
Self	21,024 (55.1%)	1,468 (51.9%)	19,556 (55.4%)	
Ambulance	16,961 (44.5%)	1,343 (47.5%)	15,618 (44.2%)	
Police	112 (0.3%)	12 (0.4%)	100 (0.3%)	
Canadian triage and acuity scale, n (%)				0.013
1 (Resuscitation)	1,523 (4.0%)	94 (3.3%)	1,429 (4.1%)	
2 (Emergent)	11,662 (30.6%)	813 (28.8%)	10,849 (30.7%)	
3 (Urgent)	19,536 (51.2%)	1,529 (54.1%)	18,007 (51.0%)	
4 (Less Urgent)	4,715 (12.4%)	352 (12.5%)	4,363 (12.4%)	
5 (Non-Urgent)	647 (1.7%)	34 (1.2%)	613 (1.8%)	
Vital signs at triage, Median IQR (25%, 75%)				
Pulse rate (beats/minute)	94 (106, 81)	95 (108, 83)	93 (106, 81)	<0.0001
Respiratory rate (breaths/minute)	20 (24, 18)	20 (24, 18)	20 (24, 18)	<0.0001
Oxygen Saturation (%)	96 (98, 94)	96 (98, 94)	96 (98, 94)	0.084
Systolic Blood Pressure (mmHg)	128 (143, 116)	129 (143, 117)	128 (143, 116)	0.060
Diastolic Blood Pressure (mmHg)	78 (86, 70)	78 (87, 70)	78 (86, 70)	0.16
Arrival temperature (°C)	36.90 (37.5, 36.5)	37 (37.5, 36.5)	36.90 (37.5, 36.5)	0.0012
COVID symptoms, n (%)				
Cough	20,557 (54.7%)	1,816 (64.3%)	19,002 (53.8%)	<0.0001
Shortness of Breath	20,443 (53.6%)	2,011 (71.2%)	18,432 (52.2%)	<0.0001
Fever	15,352 (40.3%)	1,190 (42.1%)	14,162 (40.1%)	0.037
Fatigue/Malaise	12,070 (31.7%)	928 (32.8%)	11,142 (31.6%)	0.16
Asthma Medication Type, n (%)				
Short-acting beta-agonists (SABA)	2,189 (5.7%)	884 (31.3%)	1,305 (3.7%)	<0.0001
Inhaled corticosteroids (ICS) monotherapy	1,684 (4.4%)	613 (21.7%)	1,071 (3.0%)	<0.0001
Long-acting beta-agonists (LABA)	385 (1.0%)	181 (6.4%)	206 (0.6%)	<0.0001
Combination agents (ICS/LABA)	521 (1.4%)	231 (8.1%)	290 (0.8%)	<0.0001
Triple therapy (ICS/LABA/LAMA)	153 (0.4%)	65 (2.3%)	88 (0.3%)	<0.0001
Modified Charlson Comorbidity Index Score, n (%)				<0.0001
0	11,336 (29.7%)	780 (27.6%)	11,336 (32.1%)	
1	6,153 (16.1%)	483 (17.0%)	5,373 (15.2%)	
2	4,447 (11.76%)	323 (11.4%)	3,964 (11.2%)	
3	3,359 (8.8%)	238 (8.4%)	3,036 (8.6%)	
4 +	12,844 (33.7%)	1,002 (35.5%)	11,604 (32.9%)	
Vaccination status, n (%)				0.0005
Unvaccinated	27,985 (73.4%)	1,988 (70.4%)	25,997 (73.6%)	
Partially vaccinated	738 (1.9%)	71 (2.5%)	667 (1.9%)	
Fully Vaccinated	317 (0.8%)	31 (1.1%)	286 (0.8%)	
Not documented	9,099 (23.9%)	736 (26.0%)	8,363 (23.7%)	
Outcomes, n (%)				
Hospital admission	15,431 (40.5%)	1,206 (42.7%)	14,225 (40.3%)	0.012
Intubation	1,589 (4.2%)	142 (5.0%)	1,447 (4.2%)	0.012
Death	2,733 (7.3%)	176 (6.3%)	2,557 (7.3%)	0.13
Composite Outcome	3,763 (10%)	267 (9.57%)	3,496 (10.04%)	0.43
Severe COVID-19	1,992 (5.2%)	165 (5.8%)	1,827 (5.2%)	0.13

**Table 2:** Univariate and adjusted analysis for the WHO severe COVID-19 outcome for 38139 patients presenting to Canadian ED who had documented COVID-19 infection. *Adjusted for patient-community factors (age, sex, ICS, presence of comorbidities, and vaccination status) **p-value for adjusted relative risk WHO: World Health Organization; RR: Relative Risk; ICS: Inhaled Corticosteroids; CCI: Charlson Comorbidity Index.

	Total Sample n=38139	Univariate RR	Adjusted RR*	p-value**
Exposure				
No asthma	1827/35313 (5.2%)	Ref	Ref	
Asthma	165/2826 (5.8%)	1.12 (0.96-1.32)	0.96 (0.81-1.13)	0.6377
Biological Sex				
Male	1224/19876(6.2%)	Ref	Ref	
Female	768/18263 (4.2%)	0.68 (0.62,0.74)	0.71 (0.65-0.77)	<0.0001
Age				
18-29 years	35/3998 (0.9%)	Ref	Ref	
30-39 years	112/5480 (2.0%)	2.33 (1.60-3.4)	2.25 (1.54-3.29)	<0.0001
40-49 years	231/6356 (3.6%)	4.15 (2.91-5.91)	3.72 (2.60-5.31)	<0.0001
50-59 years	346/6787 (5.0%)	5.82 (4.12-8.22)	3.77 (2.56-5.55)	<0.0001
60-69 years	439/5726 (7.7%)	8.75 (6.22-12.32)	4.52 (3.03-6.74)	<0.0001
70-79 years	400/4626 (8.6%)	9.87 (7.00-13.91)	4.65 (3.09-7.01)	<0.0001
>80+ years	429/5166 (8.3%)	9.48 (6.73-13.35)	4.45 (2.95-6.73)	<0.0001
ICS				
Nonuser	1761/36106 (4.9%)	Ref	Ref	
User	170/2033 (8.4%)	2.019 (1.73-2.34)	1.69 (1.47-1.94)	<0.0001
Charlson Comorbidity Index				
0	233/11336 (2.0%)	Ref	Ref	
1	216/6153 (3.5%)	1.70 (1.42-2.05)	1.30 (1.04-1.62)	0.018
2	240/4447 (5.4%)	2.62 (2.19-3.13)	1.74 (1.36-2.22)	<0.0001
3	222/3359 (6.6%)	3.21 (2.68-3.84)	1.80 (1.38-2.33)	<0.0001
4 +	1081/12844 (8.4%)	4.09 (3.56-4.70)	2.12 (1.64-2.75)	<0.0001
Vaccination Status				
Unvaccinated	1456/27985 (5.2%)	Ref	Ref	
Partially vaccinated	80/738 (10.8%)	2.08 (1.68-2.57)	1.53 (1.24-1.90)	<0.0001
Fully vaccinated	19/317 (6.0%)	1.15(0.74-1.78)	0.95 (0.61-1.46)	0.879
Not documented	437/9099 (4.8%)	0.92(0.83-1.02)	1.05 (0.95-1.17)	0.3372

**Table 3:** Results of unadjusted and adjusted Poisson regression measuring the association between selected covariates and the composite primary outcome of intubation or in-hospital death among patients presenting to the emergency department with COVID-19. *Adjusted for patient factors (age, sex, ICS, presence of comorbidities, and vaccination status) **P-value for adjusted relative risk ICS: inhaled corticosteroid; Ref: reference category; RR: relative risk.

Variable	Intubation or Death n/N (%)	Univariate RR	Adjusted RR*	P-value**
Asthma status				
No asthma	3377/35313 (9.6%)	Ref	Ref	
Asthma	261/2826 (9.2%)	0.96 (0.85-1.08)	0.97 (0.86-1.1)	0.68
Sex				
Male	2195/19876 (11.0%)	Ref	Ref	
Female	1443/18263 (7.9%)	0.71 (0.66-0.76)	0.71 (0.67-0.76)	<0.0001
Age category				
18-29 years	35/3998 (0.9%)	Ref	Ref	
30-39 years	105/5480 (1.9%)	2.18 (1.47-3.22)	2.07 (1.40-3.06)	0.0002
40-49 years	199/6356 (3.1%)	3.57 (2.48-5.15)	3.09 (2.13-4.48)	<0.0001
50-59 years	386/6787 (5.7%)	6.51 (4.57-9.27)	3.55 (2.38-5.29)	<0.0001
60-69 years	552/5726 (9.6%)	11.13 (7.85-15.78)	4.27 (2.85-6.41)	<0.0001
70-79 years	835/4626 (18.1%)	20.95 (14.84-29.59)	6.59 (4.38-9.91)	<0.0001
>80+ years	1526/5166 (29.5%)	34.57 (24.56-48.67)	10.54 (7.01-15.85)	<0.0001
ICS				
Nonuser	3290/36106 (9.1%)	Ref	Ref	
User	348/2033 (17.1%)	1.80 (1.62-2.01)	1.12 (1.01-1.25)	0.03
Modified Charlson Comorbidity Index Score				
0	212/11336 (1.9%)	Ref	Ref	
1	210/6153 (34%)	2.07 (1.71-2.50)	1.43 (1.12-1.82)	0.0033
2	248/4447 (5.6%)	3.33 (2.78-3.99)	1.92 (1.49-2.47)	<0.0001
3	293/3359 (8.7%)	5.17 (4.34-6.15)	2.43 (1.88-3.13)	<0.0001
4 +	2675/12844 (20.8%)	12.43 (10.82-14.28)	3.56 (2.76-4.61)	<0.0001
Vaccination Status				
Unvaccinated	2830/27985 (10.1%)	Ref	Ref	
Partially vaccinated	85/738 (11.5%)	1.13 (0.91-1.41)	0.72 (0.58-0.90)	0.003
Fully vaccinated	31/317 (9.8%)	0.95 (0.67-1.33)	0.63 (0.45-0.87)	0.0053
Not documented	692/9099 (7.6%)	0.74 (0.68-0.81)	0.94 (0.87-1.03)	0.1821

## Discussion

This study, which included more than 38,000 patients from 50 emergency departments across Canada, found no significant evidence to suggest that asthma is associated with severe COVID-19, intubation, or in-hospital death. Our data, in addition to that from prior studies, should call into question the idea that asthma is a significant risk factor for, or protective factor against, poor outcomes associated with COVID-19.

Since the beginning of the pandemic, there has been uncertainty around the relationship between asthma and COVID-19 outcomes, with conflicting theories, study results, and clinical guidelines. Early in the pandemic, recommendations from the US CDC, the WHO, and GINA identified asthma as a risk factor for more severe COVID-19 on the basis that asthma exacerbations can result from exposure to viral infections [8-10]. In contrast to these guidelines, some have advanced theories that asthma might be protective against SARS-CoV-2 infection and severe COVID-19 based on the known mechanism of SARS-CoV-2 cellular entry. The ACE-2 receptor is known to be the cellular doorway for SARS-CoV-2. Because downregulation of ACE-2 receptors occurs in patients with asthma, especially in those who use ICS, it has been hypothesized that asthma and ICS use may be protected against SARS-CoV-2 and severe COVID-19 by way of reduced viral entry into cells [19].

Prior studies examining the association between asthma and COVID-19 outcomes have provided mixed results [19-26]. For example, a study of 7,590 patients with COVID-19 from South Korea by Choi et al. aligned with our study in that no evidence for an association was identified [25]. Patients were labeled as asthmatics in this study if there were any hospital visits due to asthma symptoms or outpatient prescriptions for asthma medications in the year prior to the pandemic. The study reported that asthma was not associated with significantly higher mortality (OR: 1.31; 95% CI: 0.70-2.45) or ICU admission (OR: 0.65; 95% CI: 0.29-1.46). Despite the wide confidence intervals around the OR estimates, the authors concluded that neither a diagnosis of asthma, asthma medications nor asthma severity were independent risk factors for worse clinical outcomes of COVID-19. The main limitations of this study were related to the imprecision of the estimates and the imprecise case definition for asthma. There is a high probability that misclassification occurred based on this definition.

In contrast, a large study from the United Kingdom observed an association between asthma and COVID-19 severity. Aveyard et al. conducted a national cohort study including medical records from 1,205 general practices to examine the association of pre-existing respiratory disease in patients hospitalized with COVID-19 [22]. The study included 8,256,161 participants, of whom 14,479 (0.2%) were admitted to the hospital with COVID-19, 1,542 (0.02%) were admitted to the ICU, and 5,956 (0.1%) died. In this cohort, patients with asthma had a higher risk of hospitalization compared to those without asthma (hazard ratio (HR): 1.18; 95% CI: 1.13-1.24), and those with severe asthma had a higher risk of hospitalization compared to those with less severe asthma (HR: 1.29; 95% CI: 1.22-1.37). There was no difference in risk of death based on a history of asthma (HR: 0.99; 95% CI: 0.91-1.07). The strength of this study was its large sample size, which produced precise estimates and their ability to stratify asthma based on clinical severity, potentially improving the power to discern an association between more severe disease and COVID-19 outcomes.

Using data from the ISARIC, Bloom et al. analyzed data from 75,463 patients and noted that the association between the underlying respiratory condition and admission to hospital or death due to COVID-19 varied substantially across different age groups and use of asthma medications [26]. Similar to our study, they did not observe any association between asthma and the need for mechanical ventilation (OR: 1.17; 95% CI: 1.00-1.38); however, patients with asthma had a higher risk of requiring critical care (OR: 1.2; 95% CI: 1.05-1.37), non-invasive ventilation (OR: 1.36; 95% CI: 1.18-1.57), or oxygen (OR: 1.33; 95% CI: 1.17-1.50), compared to those without asthma. This study included only patients admitted to the hospital, likely resulting in an overall sicker population of patients compared to our study of ED patients, most of whom were managed as outpatients.

We made some other observations that are important. Despite low vaccination percentages in the cohort, we were still able to demonstrate that vaccination was less frequently associated with the primary outcome (RR: 0.63; 95% CI: 0.45-0.87) after adjusting for age, sex, ICS use, asthma status, and comorbidities. A key implication is that vaccination remains an important public health measure for all patients, including those with asthma. Moreover, our findings suggest that older age and the presence of comorbidities (i.e., the presence of multiple chronic diseases or conditions in a single individual) are significantly and independently associated with severe COVID-19 outcomes. This finding has important implications for prioritizing health care resources, as those who are older and have comorbidities should continue to be prioritized for vaccines, therapeutics, and other forms of preventive care that can mitigate the higher risk for morbidity and mortality with SARS-CoV-2 infection.

Finally, we observed that users of ICS were at increased risk of in-hospital death or intubation (aRR: 1.12; 95% CI: 1.01-1.25), as well as severe COVID-19 (aRR: 1.69; 95% CI: 1.47-1.94) compared to those who do not use ICS, even after controlling for age and presence of comorbid conditions. This adds more evidence to refute the hypothesis that ICS could be protective through downregulation of the ACE-2 receptor [27]. In line with this theory, some epidemiologic studies have observed a protective effect of the use of ICS against developing severe COVID-19 outcomes [21,27,28]. One open-label randomized controlled trial demonstrated a shorter duration of illness and reduced use of acute care resources with the use of inhaled budesonide in patients with COVID-19 [12]. One possible explanation for our divergent observation is that some of the patients in our cohort could have been misclassified as having asthma, when in fact, they had COPD [29]. Patients with COPD are also managed with ICS agents, and they are likely to experience worse outcomes with COVID-19 [30]. In this way, unaccounted COPD may have confounded the relationship between ICS use and COVID-19 outcomes.

Data from our study do not support special consideration for people with asthma in the development of policies regarding COVID-19-related public health measures, clinical management of COVID-19, or the use of antiviral therapeutics targeting SARS-CoV-2. Our study does not provide any evidence to suggest that asthma should be a consideration in vaccination prioritization, public health/infection control guidance, or risk-benefit determinations for therapeutic indications based on the independent association of asthma with risk for poor outcomes in COVID-19.

Limitations

Our study has several limitations. We may have misclassified some patients regarding their asthma status. The diagnosis of asthma is often made clinically but requires confirmation through lung function testing to optimize accuracy. Symptoms of asthma often overlap with other chronic lung conditions, including COPD. For these reasons, using the documentation of asthma in the ED or hospital records to define a diagnosis of asthma, as we did in this study, may have resulted in misclassification. Misclassification may have led to attenuated effect estimates and biased the results toward the null. Additionally, we may have missed some patient outcomes that occurred outside of hospitals participating in the study. If a patient died in the community or was intubated or died in a non-participating hospital, those outcomes would not have been accounted for in our study. We do not anticipate that this occurred more often in asthmatics versus non-asthmatics, or vice versa. There may have been some unmeasured confounders of the association between asthma and outcomes (e.g., smoking/vaping, diet, medication compliance, obesity, etc.). Future research evaluating the association between asthma and COVID-19 should consider social determinants of health such as employment, income, household crowding, gender, Indigenous and racialized status, ethnicity, and lifestyle factors such as smoking and substance use that were not available for our analysis.

Our cohort was from a relatively early phase in the pandemic when most people were not vaccinated and had not developed natural immunity against SARS-CoV-2. This may impact the generalizability of our results in relation to the present day when vaccination rates and natural immunity are very high; however, given the negative findings in our study, the likelihood of a meaningful association between asthma and COVID-19 outcomes in a population with higher immunity is very unlikely. It is possible that the relationship between asthma and COVID-19 outcomes might be different in the context of new viral variants. This will need to be explored in future research.

## Conclusions

Asthma is not significantly associated with severe COVID-19 disease, intubation, or in-hospital death among patients presenting to the ED with symptomatic SARS-CoV-2 infection. Through further research, clinicians should consider other more critical factors (e.g., comorbidities, vaccination rates, variants, and age) in decisions regarding patients infected with COVID-19.

## Appendices

**Table 4:** Characteristics of hospitals included in the Canadian COVID-19 Emergency Department Rapid Response Network (CCEDRRN). LHSC: London Health Sciences Centre; BC: British Columbia; AB: Alberta; SK: Saskatchewan; ON: Ontario; QC: Quebec; NB: New Brunswick; NS: Nova Scotia

Hospital	Province	Hospital Type	
		Rural/Urban	Teaching
Vancouver General Hospital	BC	Urban	Teaching
Lions Gate Hospital	BC	Urban	NT
Saint Paul’s Hospital	BC	Urban	Teaching
Mount St Joseph’s Hospital	BC	Urban	NT
Surrey Memorial Hospital	BC	Urban	Teaching
Royal Columbian Hospital	BC	Urban	Teaching
Abbotsford Regional Hospital	BC	Urban	NT
Eagle Ridge Hospital	BC	Urban	NT
Royal Inland Hospital	BC	Urban	NT
Kelowna General Hospital	BC	Urban	Teaching
University of Alberta Hospital	AB	Urban	Teaching
Foothills Medical Centre	AB	Urban	Teaching
Rockyview General Hospital	AB	Urban	Teaching
Peter Lougheed Centre	AB	Urban	Teaching
South Health Campus	AB	Urban	Teaching
Royal Alexandra Hospital	AB	Urban	Teaching
Northeast Community Health Centre	AB	Urban	Teaching
St Paul’s Hospital	SK	Urban	Teaching
Royal University Hospital	SK	Urban	Teaching
Saskatoon City Hospital	SK	Urban	Teaching
Sunnybrook Hospital	ON	Urban	Teaching
The Ottawa Hospital - Civic	ON	Urban	Teaching
The Ottawa Hospital - General	ON	Urban	Teaching
Kingston General Hospital	ON	Urban	Teaching
Hamilton General Hospital	ON	Urban	Teaching
Jurvinski Hospital	ON	Urban	Teaching
Health Science North	ON	Urban	NT
University Hospital - LHSC	ON	Urban	Teaching
Toronto Western Hospital	ON	Urban	Teaching
Hotel Dieu Hospital of Lévis	QC	Urban	NT
Jewish General Hospital	QC	Urban	Teaching
Centre Hospitalier Universitaire de Laval (CHUL)	QC	Urban	Teaching
Royal Victoria Hospital	QC	Urban	Teaching
Hôpital de l'Enfant-Jésus	QC	Urban	Teaching
Hôpital du Saint-Sacrement	QC	Urban	Teaching
Hôpital Saint-François d'Assise	QC	Urban	Teaching
Hôtel-Dieu de Québec	QC	Urban	Teaching
Institut Universitaire De Cardiologie Et De Pneumologie de Québec (IUCPQ)	QC	Urban	Teaching
Hôpital du Sacré-Coeur	QC	Urban	Teaching
Montreale General Hospital	QC	Urban	Teaching
Saint John Regional Hospital	NB	Urban	Teaching
Halifax Infirmary	NS	Urban	Teaching
Dartmouth General Hospital	NS	Com	NT
Hants Community Hospital	NS	Rural	NT
Cobequid Community Health Centre	NS	Com	NT
Secondary Assessment Centers	NS	Urban	Teaching

**Table 5:** CCEDRRN research ethics board approval CCEDRRN: Canadian COVID-19 Emergency Department Rapid Response Network

Province	Research Ethics Board	File Number
Alberta	University of Calgary Conjoint Health Research Ethics Board	REB20-0534
British Columbia	University of British Columbia Clinical Research Ethics Board	H20-01015
Manitoba	University of Manitoba Health Research Ethics Board	H2020:261
New Brunswick	Horizon Health Network Research Ethics Board	100890
Nova Scotia	Nova Scotia Health Authority Research Ethics Board	1025682
Ontario	Queens University Health Sciences & Affiliated Teaching Hospitals Research Ethics Board	2165
Québec	Centre intégré de santé et de services sociaux de Chaudière-Appalaches	MP-23-2021-766
Saskatchewan	University of Saskatchewan Biomedical Research Ethics Board	1935

**Table 6:** Comparison of the original Charlson Comorbidity Index to the modified Charlson Comorbidity Index used in this study. *the CCEDRRN data for this study were collected retrospectively. Comorbid events occurred prior to the index visit date, as per convention when calculating Charlson index scores. ^$^The CCEDRRN data contains coronary artery disease (CAD), and as CADs are usually a cause of myocardial infarction, we will weigh CAD as MI in the original CCI. ^#^Chronic lung disease was defined in the CCEDRRN database as any chronic lung disease except asthma and idiopathic lung fibrosis (IPF). We considered patients having asthma and pulmonary fibrosis as having a chronic lung disease and assigned a weight of 1 to each comorbidity ^€^Leukemia and lymphoma data were not collected from the CCEDRRN database. AIDS: Acquired immunodeficiency syndrome; CCI: Charlson Comorbidity Index; IPF: interstitial pulmonary fibrosis; TIA: transient ischemic attack; CCEDRRN: Canadian COVID-19 Emergency Department Rapid Response Network

Original CCI		Modified CCI*	
Comorbid condition	Weight	CCEDRRN Comorbid conditions	Weight
Age		Age	
50 – 59	1	50 – 59	1
60 – 69	2	60 – 69	2
70 – 79	3	70 – 79	3
≥80	4	≥80	4
Myocardial infarct	1	Coronary artery disease^$^	1
Congestive heart failure	1	Congestive heart failure	1
Peripheral vascular disease	1	Not available	N/A
Cerebrovascular disease	1	Stroke/TIA or seizure	1
Dementia	1	Dementia	1
Chronic pulmonary disease	1	Chronic lung disease (not asthma/IPF) or Asthma or pulmonary fibrosis^#^	1
Mild liver disease	1	Mild liver disease	1
Connective tissue disease	1	Not available	N/A
Diabetes with end-organ damage	2	Diabetes	1
Any tumor	2	Active (spread)or past malignant neoplasm	2
Leukemia	2	malignant cancer^€^	2
Lymphoma	2		
Diabetes without chronic complication	1	Not available	
Moderate/severe renal disease	2	Chronic Kidney Disease or Dialysis	2
Moderate/severe liver disease	3	Moderate/severe liver disease	3
Ulcer disease	1	Not available	
Hemiplegia	2	Not available	
Metastatic solid tumor	6	Not available	
AIDS	6	Not available	
